# The complete mitochondrial genome of *Goniurosaurus varius* (Squamata: Eublepharidae)

**DOI:** 10.1080/23802359.2023.2278817

**Published:** 2023-11-09

**Authors:** Zhengyan Zhou, Lin Ding, Ziyi Liu, Longming Fu, Lanying Xu, Lin Feng, Sufan Yu, Pipeng Li, Yu Zhou

**Affiliations:** a College of Life Science and bioengineering, Shenyang University, Shenyang, China; b Institute of Herpetology, Shenyang Normal University, Shenyang, China; c College of Life Science, Shenyang Normal University, Shenyang, China

**Keywords:** *Goniurosaurus varius*, Illumina sequencing, mitogenome, phylogenetic analysis

## Abstract

The complete mitochondrial genome sequence of *Goniurosaurus varius* is 17778 bp in length (GenBank accession number OQ992199), containing 13 protein-coding genes, 2 rRNA genes, and 22 tRNA genes. The gene order and orientation are identical to those of other Eublepharidae species in the GenBank database. Seven protein-coding genes (*COX2*, *COX3*, *ND1*, *ND2*, *ND3*, *ND4* and *CYTB*) exhibit incomplete stop codon ‘T.’ Phylogenetic analysis revealed the monophyly of *Goniurosaurus* and Eublepharidae and suggested that *G. varius is* closely related to the lineage composed of *G. luii* and *G. liboensis*. Distinct from other published Eublepharidae species, *G. varius* contains an extra non-coding region between *tRNA-Thr* and *tRNA-Pro*, which may be formed by gene rearrangements. The complete mitochondrial genome will be helpful for further studies on the population genetics of this species and phylogenetic analyses of Eublepharidae.

## Introduction

The genus *Goniurosaurus* Barbour, 1908a is a group of terrestrial eublepharid geckos that comprises 25 living nominal taxa in East Asia (Uetz et al. 2023). *Goniurosaurus varius* (Qi 2020), which is commonly named the Nanling Leopard Gecko, is a recently described new species with variable dorsal color patterns (Qi et al. 2020). *G. varius* is currently known only from the karst environment of the Nanling National Nature Reserve, northern Guangdong Province, China (Qi et al. 2020). The narrow distribution implies that *G. varius* needs more attention in conservation and evolution research. mtDNA is the main source of markers for phylogenetic research on *Goniurosaurus* (Jonniaux and Kumazawa 2008; Grismer et al. 2021), and the mt-genome sequence is still absent. Therefore, we assembled and analyzed the complete mitochondrial genome of *G. varius* for the first time. The results would be able to provide molecular data for future studies of this species.

## Materials and methods

**Figure 1.**
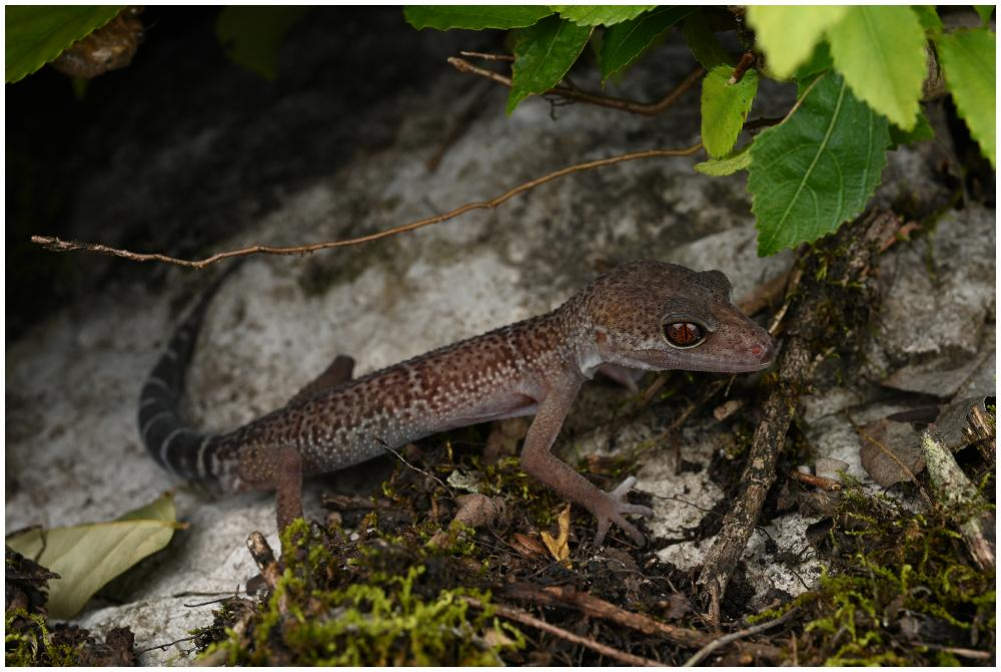
Species reference images of *Goniurosaurus varius* (Qi 2020). the stored tail tip tissue (voucher no. ZY-23042301) was taken from the specimen in this picture (Figure 2).

Adult *G. varius* were collected from Shimentai National Nature Reserve, Yingde City, Guangdong Province, China (24.780°N, 112.811°E) on 15 July 2022 (Figure 1). In lizards, toe and tail clipping is commonly used for identification and DNA analysis (Ryberg et al. 2013; Holmes et al. 2016; 2023). We took a 0.5 cm piece of tail tissue (stored in 95% ethanol) from *G. varius* for genetic analyses, and then it was released immediately after treating wounds with antiseptic. Total genomic DNA was isolated from approximately 1 mm^3^ of tail tip tissue using the TIANamp Genomic DNA Kit (TIANGEN Biotech) according to the manufacturer’s instructions. The other tail tip tissue was deposited at Shenyang Normal University, Shenyang, China (Yu Zhou is the contact person: zhouyu1988@outlook.com) under voucher number ZY-23042301. The sequencing library was prepared by Sangon Biotech, Shanghai, China and sequenced by the Illumina HiSeq 2500 platform. The mitochondrial genome was assembled *de novo* using NOVOPlasty v4.3.1 (Dierckxsens et al. 2017) with a partial mitochondrial 16S rRNA sequence of *G. varius* (GenBank: MW721829) (Grismer et al. 2021) used as the seed sequence. The average depth of coverage was generated according to the method of Ni et al. (2023). The mitochondrial genome was annotated using the MITOS Webserver (Bernt et al. 2013) with reference to the complete *Goniurosaurus luii* mitogenome (GenBank: KM455054) (Li et al. 2016). The mitochondrial genome map of *G. varius* was constructed by using Chloroplot (Zheng et al. 2020).

All six mitogenomes from the Eublepharidae species available in GenBank were downloaded as ingroup species used for phylogenetic analysis (Table 1). Two mitogenomes from the Gekkonidae species were used as outgroups (Table 1). For each of the 13 protein-coding genes (PCGs), DNA sequences were aligned using MUSCLE v3.8.31 (Edgar 2004) with the default parameters set. The alignments were further refined using Gblocks 9.1b (Castresana 2000) with the ‘codon’ model and other default settings. All refined alignments were then concatenated into the final data set. For the concatenated data set, we manually defined three partitioning strategies: unpartitioned, three partitions (one partition for each codon position), and 13 partitions (one partition for each PCG). Comparisons of the three partitioning strategies and selection of corresponding nucleotide substitution models were conducted with the Bayesian information criterion implemented in PartitionFinder (Lanfear et al. 2012). The 3-partition scheme was chosen as the best-fitting partitioning strategy, and all three partitions favored the GTR + G + I model. The phylogenetic relationships within Eublepharidae species were reconstructed using maximum likelihood (ML) analysis. Two species from the sister family Gekkonidae were included as outgroups (Figure 3). The ML tree was estimated by using RAxML version 8.0 (Stamatakis 2014) with the ‘GTRGAMMAI’ model. Support for nodes in the ML tree was assessed with a rapid bootstrap analysis (option -f a) with 100 replicates.

**Figure 2.**
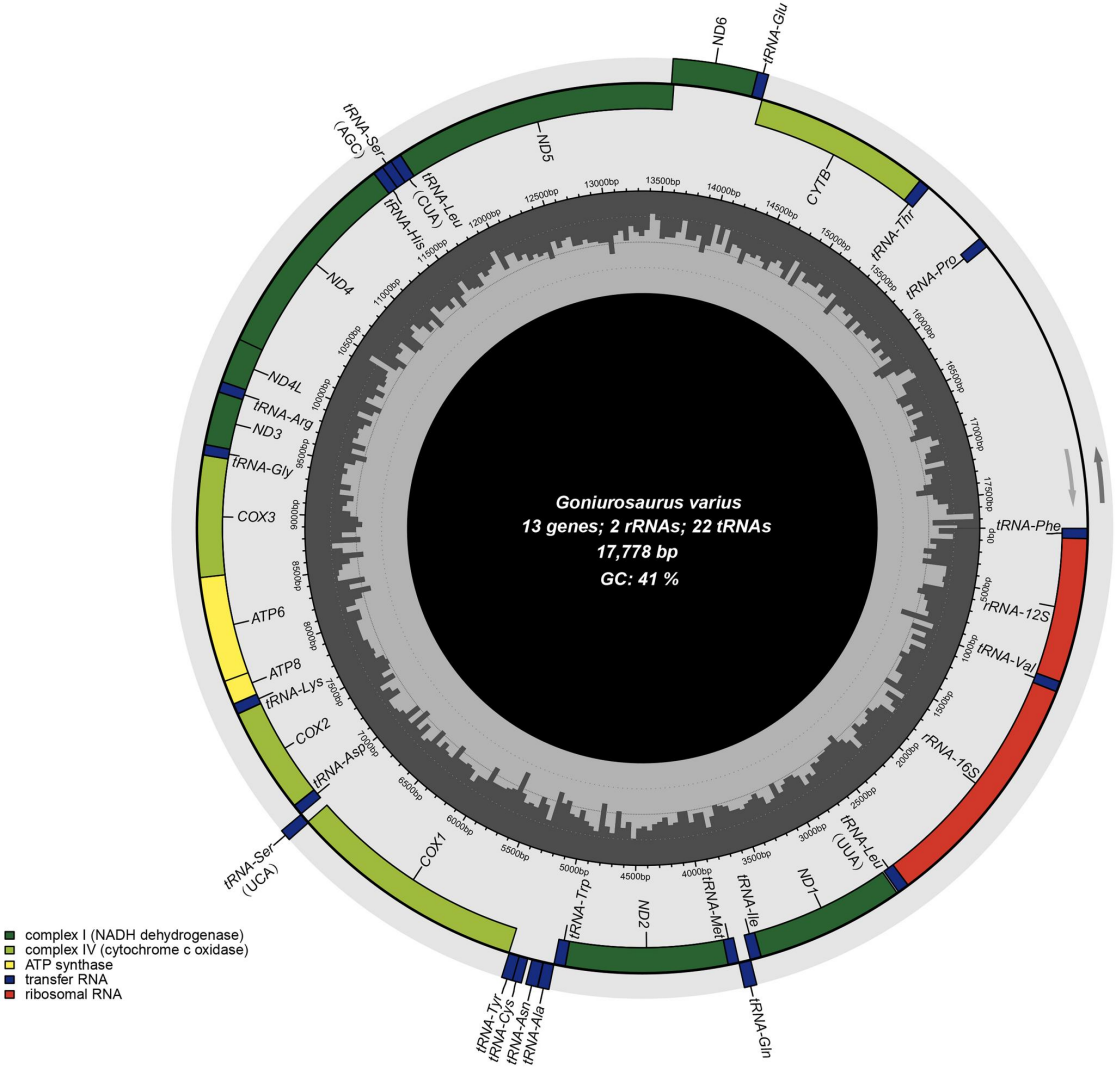
Mitochondrial genome map of *G. varius*. Arrows are the transcribed direction of genes. GC and at contents across the mitochondrial genome are shown with dark and light shading, respectively, inside the inner circle.

**Figure 3.**
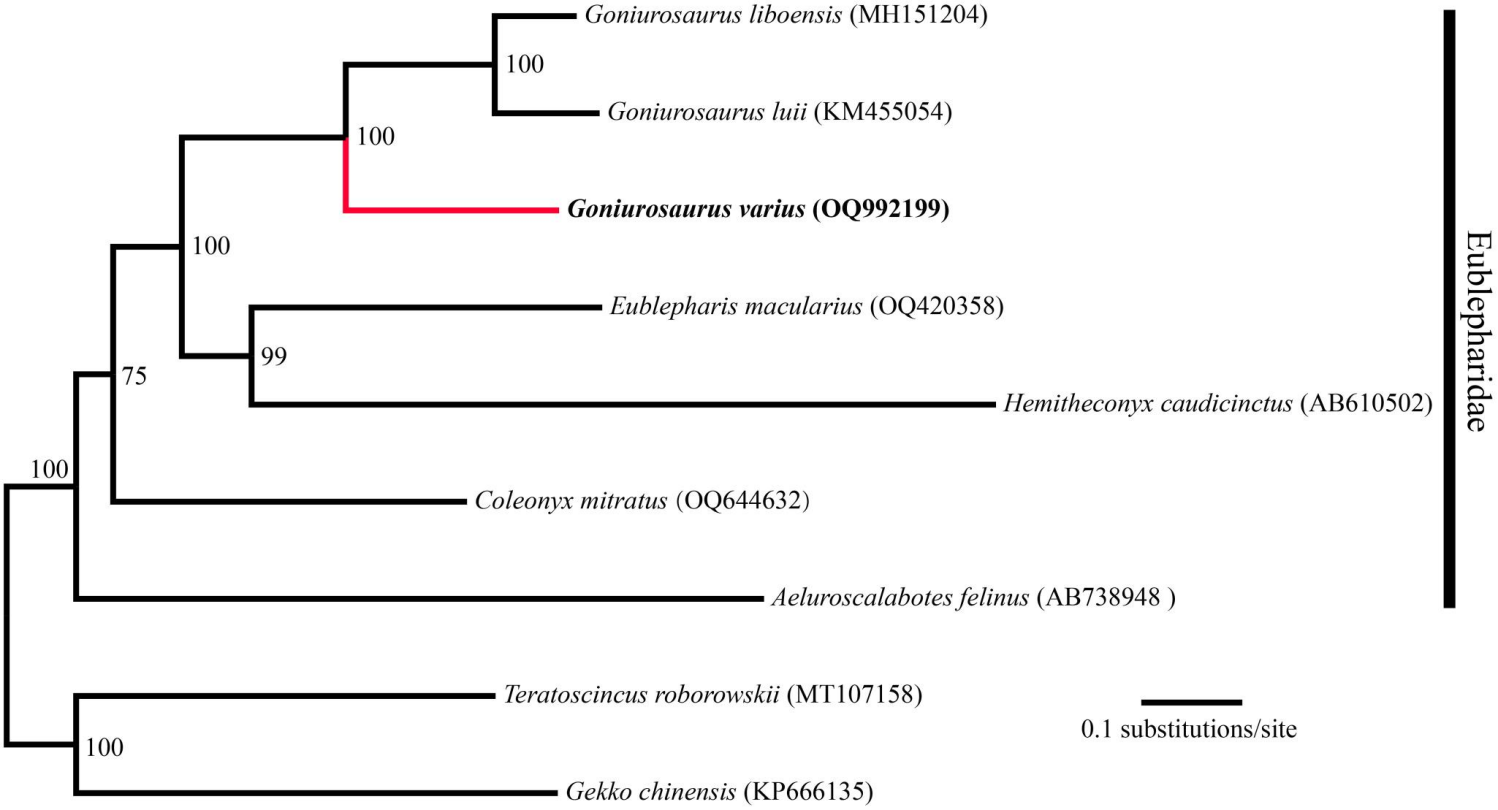
Maximum likelihood (ML)-based phylogenetic relationships of nine Eublepharidae species based on 13 PCGs.

**Table 1.** Species and GenBank accession numbers of PCGs used in this study.

Species	Accession ID				References
	Mt-genome	ND1&ND2	COX1	CYTB	
*Goniurosaurus luii*	KM455054				Li et al. 2016
*G. liboensis*	MH151204				Unpublished partial Mt-genome
*G. varius*	OQ992199				This study
*Eublepharis macularius*	OQ420358				Pinto et al. 2023
*Hemitheconyx caudicinctus*	AB610502				Jonniaux et al. 2012
*Coleonyx mitratus*	OQ644632				Lisachov et al. 2023
*Aeluroscalabotes felinus*	AB738948				Unpublished
*Gekko chinensis*	KP666135				Hao et al. 2016
*Teratoscincus roborowskii*	MT107158				Ma et al. 2020

## Results

The circular mitochondrial genome of *G. varius* was successfully assembled (OQ992199) with a length of 17,778 bp and an average coverage of 328.9 (Figure S1, supplementary material). The mitogenome of *G. varius* contains 13 PCGs, 22 tRNA genes, and 2 rRNA genes (Figure 2). The mitochondrial gene composition and order in *G. varius* are identical to those in other Eublepharidae mitogenomes (Jonniaux et al. 2012; Li et al. 2016). Seven PCGs (*COX2*, *COX3*, *ND1*, *ND2*, *ND3*, *ND4* and *CYTB*) use the stop codon of TAA. In contrast to other Eublepharidae species, the mitogenome of *G. varius* contains a 456 bp non-coding region between *tRNA-Thr* and *tRNA-Pro* (Figure 2). The nucleotide composition of this mitogenome is A 32.71%, C 27.88%, G 13.39%, and T 26.01%, with an AT-biased content of 58.72%, which is similar to the 61.5% of *Goniurosaurus luii* (GenBank: KM455054). Phylogenetic relationships among the mitogenomes of 9 Eublepharidae species were successfully reconstructed with strong support (Figure 3). Phylogenetic analysis revealed the monophyly of *Goniurosaurus* and Eublepharidae (Figure 3). The phylogenetic tree showed that *G. varius* was the most closely related to the lineage that was composed of *G. luii* and *G. liboensis*.

## Discussion and conclusions

The number and identity of the genes encoded in the mitochondrial (mt) genome are highly conserved in bilaterian animals, while the order and orientation and extra non-coding region in which they appear on the circular double-strand DNA molecule are not (Boore 1999). Gene rearrangements are known to be the main reason for mitogenome structural differentiation. In this study, *G. varius* contains a non-coding region between *tRNA-Thr* and *tRNA-Pro*, which is different from other published Eublepharidae species. We hypothesize that this extra non-coding region was formed by gene rearrangements, and similar formations were also observed in other animals (Sano et al. 2005; Kilpert et al. 2012; Kurabayashi and Sumida 2013).

The complete mitogenome of *G. varius* presented here will be useful for investigating the phylogeny and population genetics of this species, *Goniurosaurus* and even Eublepharidae.

## Supplementary Material

Supplemental Material

## Data Availability

The genome sequence data that support the findings of this study are openly available in GenBank of the NCBI at https://www.ncbi.nlm.nih.gov/ under accession no. OQ992199. The associated BioProject, SRA, and Bio-Sample numbers are PRJNA995366, SRX21042629, and SAMN36468329, respectively.
