# Supplementary material for: The complete mitochondrial genome of Goniurosaurus varius (Squamata: Eublepharidae)
Source: Mitochondrial DNA B Resour. 2023 Nov 9;8(11):1215–9. doi: 10.1080/23802359.2023.2278817 (PMC10796122; doi:10.1080/23802359.2023.2278817)
Supplement: Supplemental Material [file TMDN_A_2278817_SM6335.docx]

**Title:** **The complete mitochondrial genome of *Goniurosaurus varius* (Squamata: Eublepharidae)**

Zhengyan Zhou^1^, Lin Ding^1^, Ziyi Liu^1^, Longming Fu^1^, Lanying Xu^1^, Hongji Chen^1^, Sufan Yu^1^, Pipeng Li^2^, Yu Zhou^2^

^1^College of Life Science and bioengineering, Shenyang University, Shenyang, China

^2^College of Life Science, Shenyang Normal University, Shenyang, China

^*^Corresponding authors: Yu Zhou (zhouyu1988@outlook.com), Pipeng Li (104466606@qq.com)


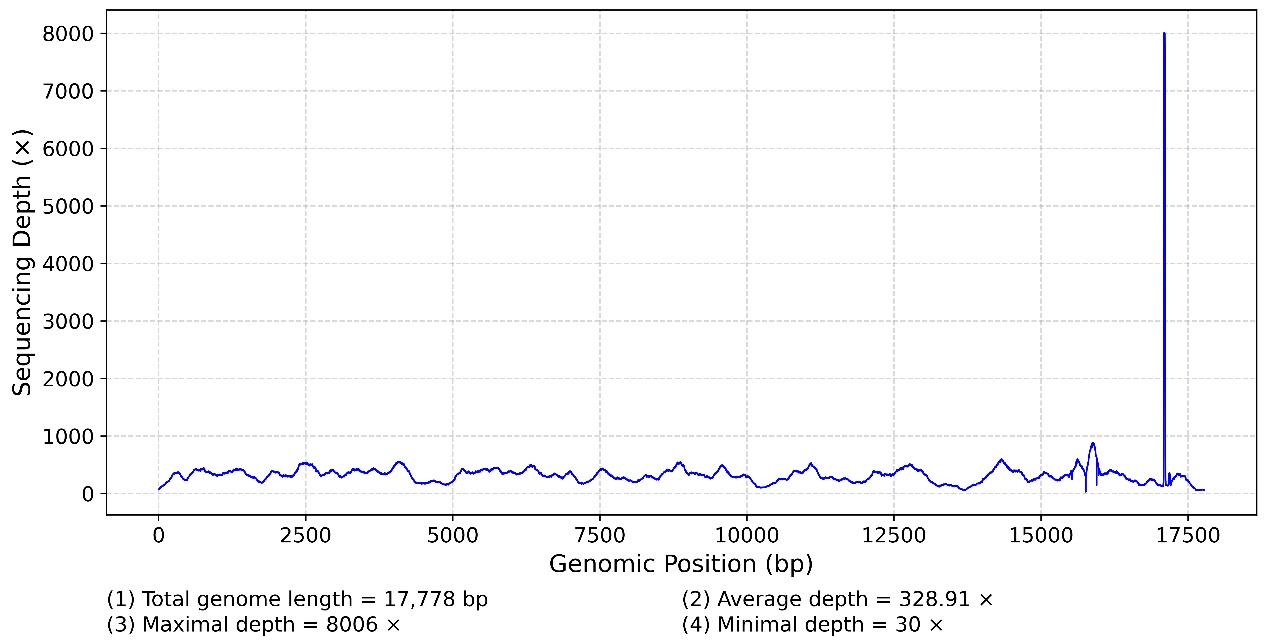


Figure S1. Sequencing depth and coverage map of the *G. varius* mitochondrial genome.
